# One-stage dorsal inlay oral mucosa graft urethroplasty for anterior urethral stricture

**DOI:** 10.1186/1471-2490-14-35

**Published:** 2014-05-08

**Authors:** Yidong Liu, Likai Zhuang, Weijing Ye, Ping Ping, Ming Wu

**Affiliations:** 1 Department Of Urology, Renji Hospital, School of Medicine, Shanghai Jiaotong University, Shanghai, China

## Abstract

**Background:**

Anterior urethral stricture remains a great challenge. We reported our clinical technique and results by using inlay dorsal buccal mucosal graft urethroplasty for repair of anterior urethral stricture.

**Methods:**

From January 2005 to July 2008, 87 male patients (range from 9 to 72 years old) with anterior urethral stricture have been treated by one-stage dorsal inlay oral mucosal graft (OMG) urethroplasty. All patients gave written informed consent for their participation. All patients showed that urethral plate had been either scarred or removed previously. In our surgery, the urethra was dissected dorsally and scar of the urethral plate was removed. The remnant urethral plate was split at midline and a buccal mucosa patch was inserted between the two parts. Neourethra was tubularized and covered with dartos flap. The pre-operative assessments included clinical data, urine analysis, uroflowmetry, retrograde and voiding cystogram, urethral ultrasonography and endoscopy. Postoperatively, the flow rate and void residual volume were analyzed by uroflowmetry and sonography. Any further instrumentation to assist voiding was considered as failure.

**Results:**

After 12 to 48 months (mean: 25.8 months), 78 patients (89.66%) have shown good results by the one-stage urethroplasty. However, 9 patients (10.3%) required further treatment due to recurrence, while 6 patients (6.9%) had fistula.

**Conclusions:**

This one-stage dorsal inlay approach using dorsal oral mucosal grafts is a reliable method to create a substitute urethral plate for tubularization. This approach represents a safe option for anterior urethral stricture patients especially with grossly scarred urethral plate.

## Background

Successful repair of anterior urethral stricture remains a challenging issue, particularly in patients following previous surgical attempts. The most important causes of anterior urethral stricture are idiopathy, transurethral resection, urethral catheterization, and previous hypospadias surgery [1]. Without treatment, urethral stricture can result in urinary tract infection and acute urinary retention, which could secondarily lead to bladder thickening, irritability and urinary fistula with gangrenous inflammation. The urethroplasty is more difficult in patients with previous surgeries [2], since the formation of spongiofibrosis or scar tissue and poor blood supply. It has been reported that long-term recurrence rate is up to 31.6% after previous surgery [3]. However, ultimate reconstruction should still be attempted by further urothroplasty.

Free extragenital graft tissues such as the ureter, saphenous vein, appendix, full-thickness skin, bladder mucosa, buccal mucosa, and lingual mucosa [4,5] have been used for urethral reconstruction. Among those grafts, it has been currently accepted that buccal mucosa is one of the best options, which possesses the advantages of constant availability, easy harvesting, favorable immunological properties (resistance to infection) and tissue characteristics (a thick epithelium, high content of elastic fibers, thin lamina and rich vascularization) [1,6-10]. The dorsal inlay approach for anterior urethroplasty has been widely used due to its safety and efficacy. The reasons are not only underlying corpora could provide better mechanical support and blood supply to the graft, but also the dorsal graft inserted into the remaining urethral plate could enlarge the diameter of new urethra. In this report, the technique of inlaying buccal mucosa graft used for repairing anterior urethral stricture is introduced and the clinical results and complications are evaluated.

## Methods

### Patients

**Figure 1:**
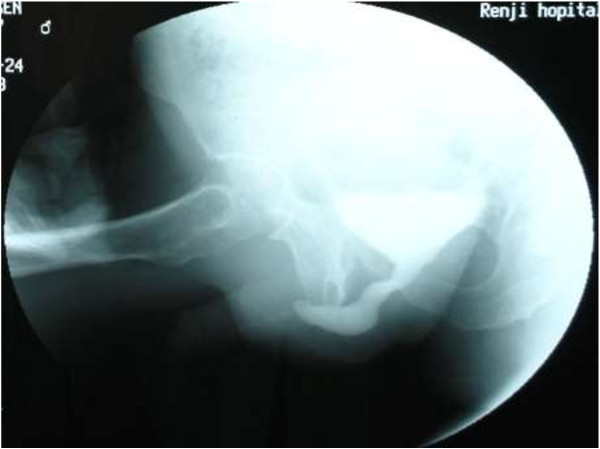
Routine preoperative imaging: a retrograde urethrogram showing a long AUS.

From January 2005 to July 2008, 87 patients (age 9–72 years old; mean 35.6 years old) underwent one-stage dorsal inlay urethroplasty with buccal mucosal graft for anterior urethral stricture. All the cases were performed by a single surgeon (YWJ) and this technique represents standard care at our hospital. The pre-operative assessments included clinical data, urine analysis, uroflowmetry (range from 0.0 ml/s – 13.5 ml/s; mean 5.6 ± 1.5 ml/s), retrograde/voiding cystogram (Figure 1) and urethral ultrasonography to evaluate the degree of scar, and endoscopy to observe the color and luster of urethral mucosa. All-mentioned examination could help to evaluate the degree scar, and the direct observation in surgery is crucial. In cases with severe scar or local infection or poor tissue blood supply, which could affect OMG survival, it is a wise choice to remove the severe scar at the first stage procedure, and convert one-stage to two-stage procedure.

All patients gave written informed consent for their participation. This study was approved by the Ethics Committee of Renji Hospital, Shanghai Jiaotong University School of Medicine and conducted in accordance with Declaration of Helsinki.

After surgery, all patients were followed up at least 12 months. Clinical evaluations including original diseases, sites and length of the strictures are summarized in Table 1. 61 patients (71.26%) had a previous surgical intervention (9 cases of urethral dilatation; 15 cases of direct visual internal urethrotomy (DVIU); 12 cases of meatotomy; and 25 cases of urethroplasty).

**Table 1:** Clinical evaluations of original diseases, sites and length of the strictures

		**Number**	**Percentage (%)**
Etiology:	Inflammatory	27	31.03
	Traumatic	15	17.24
	Iatrogenic	40	45.98
	Idiopathic	5	5.74
Site of stricture:	Bulbar urethra	20	22.99
	Penile urethra	57	65.52
	Bulbar and penile urethra	10	11.49
Length of stricture:	1.0–3.0 cm	16	18.39
	3.1–5.0 cm	39	44.83
	5.1–7.0 cm	30	34.49
	7.1–10.0 cm	2	2.30
Mean stricture length			
Bulbar urethra	2.3 cm	20	22.99
Penile urethra	5.2 cm	57	65.52
Bulbar and penile urethra	7.3 cm	10	11.49

### Inlay dorsal buccal mucosal graft urethroplasty

**Figure 2:**
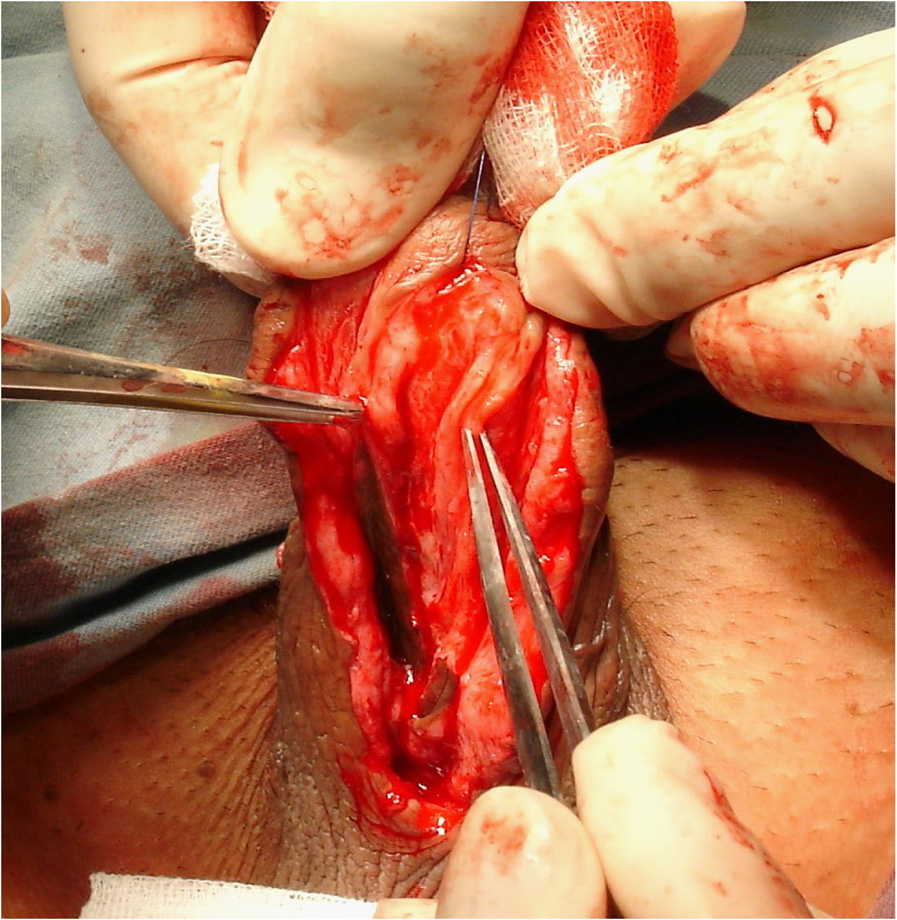
Panurethral stricture: urethra was laid open and split to the corpora cavernosa in the midline.

**Figure 3:**
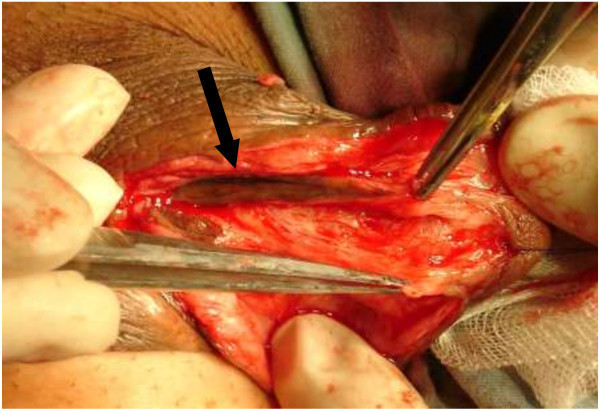
**Scar tissue was removed from the underlying corpora cavernosa, providing a supple graft bed.** (Black arrow: Previous surgery residual skin tube urethra).

**Figure 4:**
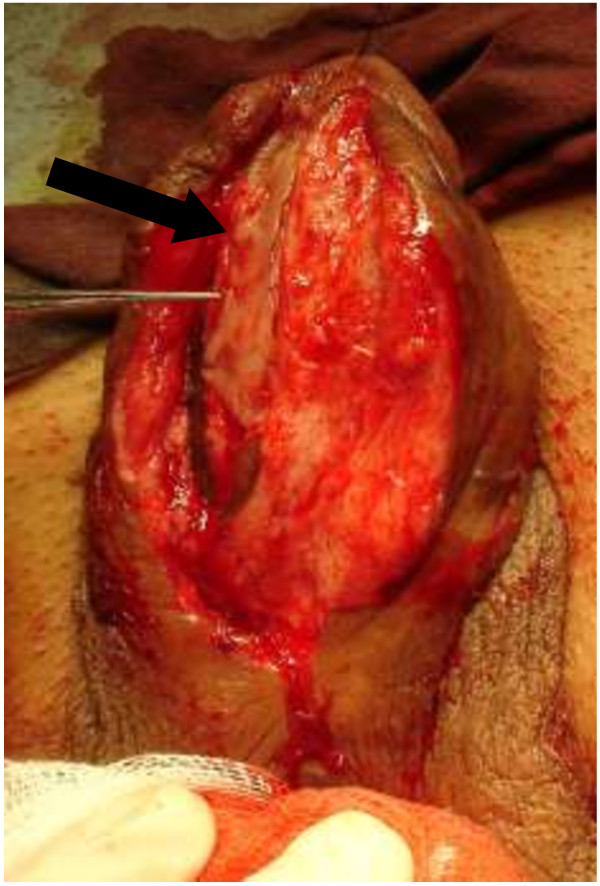
**Buccal mucosa graft tailored about 1.5 cm wide and 5.5 cm long is positioned on dorsal part of opened urethra was inserted between split urethra and quilted to the underlying corpora cavernosa.** Black arrow: The inserted buccal mucosa graft.

We performed a standard OMG (from lower ) lipharvesting technique [11].For patients with bulbar strictures, a midline perineal incision was made after the induction of dilator; while for those with penile strictures, a subcoronal circumferential incision was made. Then the penis was degloved. The urethra was split ventrally to expose the stricture. The incision was extended to the normal tissue of urethra, which was about 1-2 cm both proximally and distally. Pre-existing scars between the urethrotomy lines were removed if possible, and then the urethral plate was split and incised to the surface of tunica albuginea to create the bed for graft (Figure 2). The length of the urethrotomy was measured for harvesting an adequate free graft. Inlaid grafts were 3.0-7.5 cm in length (mean 4.59 ± 2.16 cm) and 0.9-2.2 cm in width (mean 1.26 ± 0.53 cm). The full-thickness mucosal graft was harvested mainly from the inner side of lower lip. If the graft is not long enough, cheek mucosa could be used following stripping off the fat of subcutaneous tissue. The tailored buccal mucosa graft was inserted between the split urethral plates (Figure 3). The edges of incised urethra were stitched with interrupted sutures of 6–0 or 7–0 PDS to reduce tension locally and maintain diameter normally (Figure 4). The edges of the augmented urethral plate were then closed using 6–0 or 7–0 PDS sutures with an indwelling catheter (Fr 8–16 feeding tube). Subcutaneous tissue was placed over the neourethra as a barrier layer. The penile shaft and scrotal skin was closed by “Z” shape suturing to prevent wound contracture and fistula. The consent was obtained from the patient for his participation to publish Figures 1, 2, 3 and 4.

In patients with unexpected severe scar during surgery, we performed a two-stage urethroplasty. In the first stage the urethral plate was removed and local flap was splayed and quilted over the tunica albuginea. After 6 month we performed inlay dorsal OMG urethroplasty.

The catheter was removed two weeks after operation. Postoperatively, all patients were examined by uroflowmetry in 3, 6, and 12 month, respectively.

## Results

Success was defined as no further surgical interventions such as dilatation or optical urethrotomy. The voiding flow rate should be greater than 15 ml/second, post-void residual urine less than 50 ml. If voiding flow rate less 15 ml/sec and/or post - void residual volume (PVR) more than 50 mL. We performed further investigation by cystoscopy to confirm. All patients were followed up from 12 to 48 months (mean: 25.8 months). 8 patients had Qmax < 15 ml/sec, and of these, 3 actually were found to have recurrent stricture. (ranged from 12.0-14.5 ml/s, mean: 13.0 ± 1.0 ml/s, and volume of post-void residual urine ranged from 51.0-53.0 ml, mean 52.0 ± 1.0 ml) and 5 patients with complaint of difficult urination and prolonged voiding (ranged from 16.0-18.0 ml/s, mean: 16.0 ± 0.5 ml/s, and volume of post-voiding residual urine ranged from 40.0-49.0 ml, (mean 44.0 ± 1.0 ml, Table 2). 8 patients underwent cystourethroscopy, including 3 patients with recurrent stricture and 5 patients with complaint of difficult urination and prolonged voiding. The majority of grafted mucosa survived (85/87). In 2 failed cases, the distal end of grafted mucosa did not survive, which caused recurrent stricture.

**Table 2:** Results of uroflowmetry

**Results of uroflowmetry (ml/s)**	**Numbers**	**Value ranges (ml/s)**	**Mean (ml/s)**
Pre-operative	87	0.0^a^–13.5	5.6 ± 1.5
Post-operative			
< 15.0	3^b^	12.0–14.5	13.0 ± 1.0
> 15.0	84	16.0–22.0	18.0 ± 2.2

^a^Cases under cystostomy.

^b^3 cases with **recurrent** stricture.

The overall successful rate was 89.66% (78/87). Fistula was observed in 6 patients (6.9%) 3 weeks post-operatively, 2 patients were cured spontaneously within 3 months and 4 out of them were repaired lately with rotation of a local dartos flap. The stricture recurred in 3 patients (3.45%). All of them had history of multiple surgical interventions for hypospadias repair. According to our analysis, the cause of fistula could be infection, previous surgical intervention and poor blood supply. All cases with fistula occurred in hypospadias group. Preoperative antimicrobial susceptibility, urinary diversion, postoperative use of antibiotics could reduce the rate of urinary fistula. One of them had a dilation 3 months after urethroplasty, and the recurrent stricture was cured (voiding flow rate ≥ 15 ml/sec). The other 2 cases of recurrent were solved by second one-stage dorsal inlay buccal mucosa graft urethroplasty.

All patients showed slight oral discomfort at the donor site in first two days postoperatively. They resumed normal diet 1 day after surgery. No other complications were found at the donor site following 6 months of surgery.

## Discussion

The etiology of “anterior urethral stricture” includes trauma, infection, iatrogenicity and idiopathia, and it recurs after internal urethrotomy, dilatation, or anastomotic urethroplasty. Management of anterior urethral stricture disease has been evolved over the past decade because of innovative applications of plastic surgical techniques, resulting in improved long-term outcomes. There is still no single technique for all lesions clinically. Although endoscopic treatments such as urethral dilation and optical internal urethrotomy can transiently improve urinary flow, repeated instrumentation could exacerbate scar formation increasing length and severity of stricture and complicating subsequent reconstruction. Dilation in patients with a short stricture can be an option temporarily; however, the repeated procedures should be avoided since the results are inefficient. Open urethroplasty has been currently reported as a gold-standard treatment of resistant urethral stricture disease [12,13].

Reconstructive surgery for anterior urethral stricture has been developed and modified rapidly. Despite extensive research, there is no general recommendation or guideline available for anterior urethral stricture patients. The difficulties of treatment in these patients are absence of local penile skin and formation of severe scar in the urethral plate. Consequently, fresh and supple tissue needs to be imported for augmenting the urethral plate and tubularizing a urethra [3]. Excision of stricture and repair of urethra are appropriate only for short stricture or primary lesion in patients following a blunt perineal trauma [14]. The use of flaps or grafts is mandatory in patients with long and complex strictures.

Another technique is the use of a graft adopted from the ventral surface of the urethra. However, the graft often lacks the mechanical support from fixed bed, which tends to be twisted easily resulting in the reduction of neovascularization and decrease of the caliber of the reconstructed urethra [15]. Moreover, sacculation at the graft side might also occur, which causes post voiding dribbling and ejaculatory failure [16]. Sequestration of semen and residual volume of infected urine inside the pseudo-diverticulum may further compromise the adjacent urethra and facilitate recurrent stricture disease.

Snodgrass described the tubularized incised plate (TIP) hypospadias repair in 1994 as a technique to augment the graft and improve mobilization of the urethral plate when performing a Thiersch-Duplay urethroplasty [17]. This maneuver allows constructing a new urethra with existing urethral plate. It has been suggested that healing may occur through reepithelialization in the relaxing incision without obvious scarring, allowing the incised edges to remain separated. Currently TIP urethroplasty has become a preferable method as its suitability, simplicity and efficiency. However, this technique is not applicable in patients with anterior urethral stricture, whose urethral plate is absent or severely scarred. In these patients, it is impossible to widen the caliber for reconstructing the new urethra. Therefore, the modified technique of TIP by using inlayed patch has been developed. The satisfied result has been reported by using dorsal buccal mucosa graft with urethral plate incision in hypospadias salvage [18]. Previous study from our center had also shown that use of combined procedures with the TIP technique is a reliable technique for redo hypospadias, which can replace the staged procedures [19]. We have suggested that the technique is practically available for anterior urethral stricture besides hypospadias.

The present results show that in the presence of a viable urethral plate, a one-stage dorsal inlay buccal mucosal graft urethroplasty can be successful for reconstructing all segments of the anterior urethra, even if the urethral plate is severely scarred or partly removed. We found that the approach is anatomically easier to achieve than the approach from ventral side. The approach also requires less extensive exposure of the spongy tissue, less bleeding from the corpus spongiosum, and less mechanical damage to the graft [20-22]. The dorsal onlay technique of Barbagli and the dorsal inlay technique of Asopa buccal mucosal graft urethroplasty provide similar success rates, but the Asopa technique is easy to carry out, provides shorter operative time and less blood loss, and it is associated with fewer complications for anterior urethral stricture repair [23].

A serious complication of free graft urethroplasty is necrosis of the patch induced by vascularization failure from its bed. When this occurs in ventrally placed grafts, a urethra-perineal fistula of considerable size is inevitable. We have not had this complication with patients treated by dorsal graft apposition.

Dorsal inlay graft urethroplasty is a versatile procedure, which could be combined with various substitute materials. Candidate tissues included in split and full-thickness skin grafts derived from the scrotum, penis and extragenital areas, bladder mucosa and buccal mucosa. Initially scrotal skin was used, but its long-term efficacy has been proved unacceptable [24]. Although full-thickness skin grafts have shown satisfied results, the donor-site problems might exist such as recurrent stricture. Because of its theoretical disadvantage and much long-term complication, the initial enthusiasm of using bladder mucosa declined quickly.

Oral mucosa has been known as an ideal substitute for the urethra, which include in easy accessibility with a wet environment, a thick epithelium and a thin lamina propria. Oral mucosa also demonstrates more resistance to mechanical weakening, reduction of pseudo-divertiuclum formation and early inosculation. All those advantages are comparable with full-thickness skin grafts [7].

Serious complications resulting from harvesting a OMG have been rarely reported. Possible adverse effects of harvesting OMG include intraoperative haemorrhage, postoperative infection, pain, swelling, damage to the parotid duct, limitation of oral opening and numbness through nerve damage. Comparing the outcome clinically, there was no much difference between the grafts harvested from cheek or lower lip. We prefer to use the graft from lower lip which shows less negative effects during mastication.

The successful management of urethral strictures depends on multiple factors including patient’s indication, approach’s selection and surgical skill. We suggest the following techniques in the reconstruction of neourethra. (1) The OMG should be thin and without fat layer. It will be easier for the graft to survive after implantation and also decrease the oral complication rate, such as bleeding, scar formation. (2) The scar of the urethral plate should be excised down to tunica albuginea to provide healthy recipient bed for the OMG. (3) The length of graft should surpass the distal and proximal position of stricture and sutured to healthy urethra to avoid the recurrence of stricture. However, the limitation of the approach is that stricture excision is frequently required in patients with long and dense strictures with extreme spongiofibrosis. Hence, a two-stage procedure has been developed in these unfavorable cases. The graft should be comprised primarily and then tubularizated secondarily at 6 months. In most anterior urethral stricture cases, dorsal inlay buccal mucosa grafting and subsequent urethral reconstruction can be finished in one stage.

## Conclusions

Reconstructing the urethra for anterior urethral stricture is still a challenge for urologists. Inlay buccal mucosa graft with TIP technique has both the advantages of OMG and Snodgrass technique. This technique is easier, requires less operating time and is a valuable option for most cases of anterior urethral stricture.

## Competing interest

The authors declare that they have no competing interests.

## Authors’ contributions

YL conceived of the study, and participated in its design and coordination. LZ participated in the design of the study. WY performed the surgery and helped to draft the manuscript. PP and MW performed the statistical analysis. All authors read and approved the final manuscript.

## Pre-publication history

The pre-publication history for this paper can be accessed here:

http://www.biomedcentral.com/1471-2490/14/35/prepub
